# Detailed Calorimetric Analysis of Mixed Micelle Formation from Aqueous Binary Surfactants for Design of Nanoscale Drug Carriers

**DOI:** 10.3390/nano11123288

**Published:** 2021-12-03

**Authors:** Ádám Juhász, László Seres, Norbert Varga, Ditta Ungor, Marek Wojnicki, Edit Csapó

**Affiliations:** 1 MTA-SZTE Lendület “Momentum” Noble Metal Nanostructures Research Group, Interdisciplinary Excellence Center, Department of Physical Chemistry and Materials Science, Faculty of Science and Informatics, University of Szeged, Rerrich Béla Square 1, H-6720 Szeged, Hungary; juhaszad@chem.u-szeged.hu (Á.J.); sereslaci8@gmail.com (L.S.); vargano@chem.u-szeged.hu (N.V.); ungord@chem.u-szeged.hu (D.U.); 2 MTA-SZTE Biomimetic Systems Research Group, Department of Medical Chemistry, University of Szeged, Dóm Square 8, H-6720 Szeged, Hungary; 3 Faculty of Non-Ferrous Metals, AGH University of Science and Technology, Mickiewicza Ave. 30, 30-059 Krakow, Poland; marekw@agh.edu.pl

**Keywords:** mixed micelle, calorimetry, solubilization, drug delivery

## Abstract

While numerous papers have been published according to the binary surfactant mixtures, only a few articles provide deeper information on the composition dependence of the micellization, and even less work attempts to apply the enhanced feature of the mixed micelles. The most important parameter of the self-assembled surfactants is the critical micelle concentration (*cmc*), which quantifies the tendency to associate, and provides the Gibbs energy of micellization. Several techniques are known for determining the *cmc*, but the isothermal titration calorimetry (ITC) can be used to measure both *cmc* and enthalpy change (Δ*_mic_H*) accompanying micelle formation. Outcomes of our calorimetric investigations were evaluated using a self-developed routine for handling ITC data and the thermodynamic parameters of mixed micelle formation were obtained from the nonlinear modelling of temperature- and composition- dependent enthalpograms. In the investigated temperature and micelle mole fractions interval, we observed some intervals where the *cmc* is lower than the ideal mixing model predicted value. These equimolar binary surfactant mixtures showed higher solubilization ability for poorly water-soluble model drugs than their individual compounds. Thus, the rapid and fairly accurate calorimetric analysis of mixed micelles can lead to the successful design of a nanoscale drug carrier.

## 1. Introduction

Surfactants have been part of our everyday lives for nearly two thousand years, and have been an increasingly used family of compounds in our modern society since the Industrial Revolution [1]. Due to their unique molecular structure, in addition to their commonly known detergent properties, they are widely used as solubilizing [2], stabilizing [3] and emulsifying [4] industrial chemicals and nanomaterials, yet more than half of the amount produced goes to households as a detergent. Enormous amounts of surfactants are produced globally year by year; in 2007 over 3 million tons were produced in Western Europe alone [5]. These surfactants go down the drain and into the water treatment facilities for processing [6]. Despite international regulations to protect our environment, their transport into aquatic life remains a continuous risk, so reducing the quantity produced through applied surfactants is one of today’s important technological challenges [7,8].

Development of environmentally friendly and sustainable technologies requires a comprehensive knowledge of the physicochemical rules that determine the solution and interfacial behavior of surfactants [9]. Much of this knowledge is available in the form of accepted and proven regularities, but due to the extremely diverse use and structural diversity of organic compounds, research to understand the properties of surfactants is still ongoing [10,11,12]. Of these, the study of the co-presence of surfactants with different chemical structures is a separate topic. The formation of mixed micelles, described as an association of two different surfactant molecules, has been a studied phenomenon for decades [13,14], but those physicochemical rules which can describe their formation (*cmc* value of mixed surfactant systems) and composition (molar fraction in the mixed micelles) are still undetermined. While calorimetric studies of the formation of surfactant micelles formed by individual molecules have been reported by many researchers [15], significantly fewer studies deal with micelle formation in mixed surfactant solutions [16,17]. In contrast, studies that look for a relation between the formation (and composition) and practical applicability of mixed micelles, either theoretically [18] or experimentally [19], are much less common in the literature.

However, mixed micelles of surfactants in aqueous solutions also have varied industrial application (food-, pharmaceutical-, petrochemical industry etc.) [19,20,21,22,23]. When there are adequate synergistic interactions between the individual components of micelles the critical micelle concentration (*cmc*) of the binary surfactant mixture can be lower and binary mixed micelle is thermodynamically more stable than the single surfactant containing micelle [24]. Deeper understanding of the origin of non-ideal and synergistic behavior may help to design more efficient surfactant mixtures, and in this way reduce the amount of the applied chemicals.

Precise and detailed exploration of this phenomenon is crucial as it may provide deeper information about the synergetic effect in the mixed micelle formation. For this aim, this paper demonstrates a systematic approach by analysis of quantitative calorimetric data for the characterization of aqueous associated colloids from mixed surfactants. Two surfactants with different chemical structures were selected for the experiments where their individual and mixed micelle formation were characterized by ITC method. Based on the results of the calorimetric studies, we tried to determine and analyze the value of the thermodynamic parameters of the formation of mixed micelles as functions of the composition of the bulk phase. Besides, a solubilized amount of a poorly water-soluble model drug was tested by an acidimetric method for finding a relationship between solubilization capability and composition of the mixed micellar system.

## 2. Materials and Methods

Nonionic- (2-[4-(2,4,4-trimethylpentan-2-yl)phenoxy]ethanol/Triton X-100, hereinafter denoted by TX) and cationic (N,N,N-Trimethylhexadecan-1-aminium bromide/CTABr, hereinafter denoted by CT) surfactants and other chemicals such as benzoic acid (benzenecarboxylic acid/BAc), sodium hydroxide (NaOH) and phenolphthalein (3,3-Bis(4-hydroxyphenyl)-2-benzofuran-1(3H)-one/phph) were purchased from Sigma-Aldrich Hungary Ltd., Budapest, Hungary. The surfactant solutions, their equimolar mixture-containing solutions and alkali measuring solution for acid–base titrations were prepared in a 100-mL volumetric flask and then diluted in deionized water (18 MΩ cm^−1^ Milli Q, Millipore, Burlington, MA, United States) to the desired concentration. All the starting materials were used without further purification. A syringe filter with a pore diameter of 0.45 µm (Millex-HA mixed cellulose esters (MCE) membrane) was used to filter the saturated and solubilized benzoic acid and surfactants containing colloid systems.

Thermometric titration experiments were performed with a computer-controlled VP-ITC (PTC Ltd., Mosonmagyaróvár, Hungary) power-compensation micro calorimeter (MicroCal) at 293.15, 298.15, 303.15, 308.15 and 313.15 K to determine the *cmc* (mM) and Δ*_mic_H* (kJ∙mol^−1^) of the nonionic- and cationic surfactants and their mixtures. During the calorimetric titrations, the sample cell was filled with 1.4 mL deionized water, and it was titrated under constant stirring with 300 μL of surfactant solution in aliquots of 10 μL in periodic time intervals of 5 min. The enthalpograms (calorimeter power signal vs. time) were evaluated by means of Origin Microcal 7.1. software (PTC Ltd., Mosonmagyaróvár, Hungary). The extracted enthalpograms (enthalpy of injection per mole of injected surfactant vs surfactant concentration at sample cell) were successfully described by using a modified version of Boltzmann equation [25,26] which has been used to improve the precision of the determination of the characteristic parameters (*cmc*, Δ*_mic_H*).

## 3. Results and Discussion

During the isothermal titration calorimetric (ITC) measurements, the thermal effect accompanying the dissociation of the individual micelles (CT and TX) was recorded in the solutions of the separate surfactants (*θ_TX-_*_100_ = 0.0 and 1.0) and their mixtures (*θ_TX-_*_100_ = 0.2; 0.4; 0.6 and 0.8) as a function of time. As an intermediate note, the molar fraction in the mixture (*θ*_1_ and *θ*_2_) is not the same as the molar fraction describing the whole system (*n*_1_, *n*_2_ and *n*_3_), see the detailed description in Appendix A.

### 3.1. Micelle Formation of Individual Surfactants

**Figure 1:**
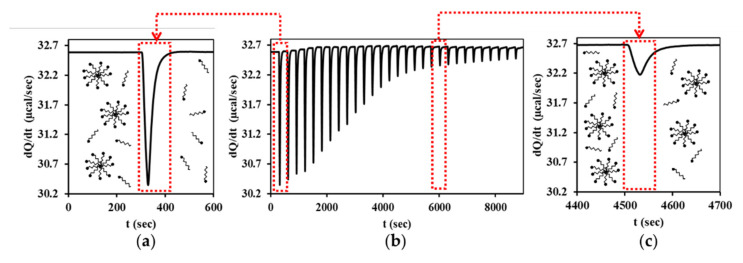
ITC raw data for the titration of nonionic surfactant (TX) at 25 °C in the middle (**b**) of the graph and a schematic representation of the calorimetric signals and processes characteristic of the pre- (**a**) and post-micellar (**c**) phases.

For successful ITC measurements, the sample dosing syringe of the device contained a surfactant solution with a concentration of about 8–10 times higher than actual cmc. The calorimetric curve shown in the middle of Figure 1 was recorded during the addition of the nonionic surfactant (TX) to a measuring chamber filled with deionized water. When a surfactant solution with a concentration greater than cmc is added to deionized water, the micelles initially dissociate into their monomers, as can be seen to the right (a) side of Figure 1. The largest calorimetric signals (heat flux: *dQ*/*dt*) can be measured as a function of time in this pre-micellar range. By further sample addition, a transition range (b) is reached where the concentration of surfactant in the measuring cell is already high enough to avoid the dissociation of micelles. Finally exceeding cmc (post-micellar phase) only the dilution of the association colloid can be monitored, as shown in (c) part of Figure 1 schematically.

**Figure 2:**
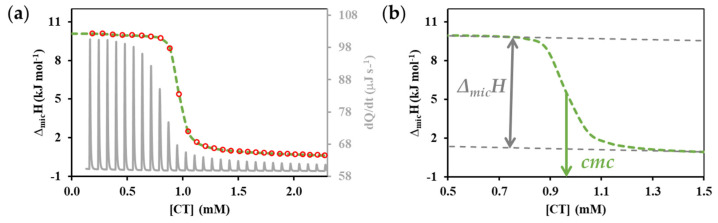
(**a**) Measured ITC raw data (grey continuous curve) and enthalpogram (red circles) for the titration of ionic surfactant (CT) at 298 K where the dashed green curve indicates the calculated enthalpogram; (**b**) The sigmoidal Boltzmann equation-based model curve of the measured enthalpogram with a schematic illustration of the parameters (Δ*_mic_H* and *cmc*) that can be calculated from the nonlinear parameter estimation.

For the quantitative evaluation of the measurement results, the enthalpy changes for the given dilution state and in our case for the dissociation process must be calculated from the differential peaks of the calorimetric signal sequence (*dQ*/*dt* vs. *t*) shown in the middle part of Figure 1 for each dosing step. The value of the enthalpy change (Δ*_mic_H*) corresponding to the area of each peak in relation to the amount of surfactant in the injected solution is given in Origin Microcal 7.1. software, calculated during an appropriate integration process. This calculation step provides the typically sigmodal enthalpograms (Δ*_mic_H*/kJ∙mol^−1^ vs. [surfactant]/mM) of the ITC procedure shown in Figure 2, which can be evaluated using nonlinear parameter estimation method, which is presented through the example of the other surfactant (CT) component. The x coordinate of the inflection points of the sigmoidal curve (the corresponding concentration value on the x-axis) provides the *cmc* value and the difference in enthalpy values characterizing the pre- and post-micellar range determines the magnitude of the enthalpy change (Δ*_mic_H*) attributable to micelle formation, as shown in part (b) of Figure 2. The Boltzmann equation based method [27,28] of calculating provides two fundamental parameters (*cmc* and Δ*_mic_H*) which are described in detail by the Supplement Materials through the evaluation of the enthalpogram of the nonionic component. Based on the nonlinear regression estimated coefficients of the calculated enthalpograms, the *cmc* of the ionic surfactant (Figure 2b) was found 0.964 ± 0.005 mM and the enthalpy change attributable to exothermic micelle formation was Δ*_mic_H^0^* = −9.16 ± 0.77 kJ mol^−1^, for the nonionic (TX) compounds (Figure S1b) the *cmc* was observed 0.319 ± 0.003 mM and endothermic micelle formation corresponds to an enthalpy of 6.96 ± 0.72 kJ mol^−1^.

**Figure 3:**
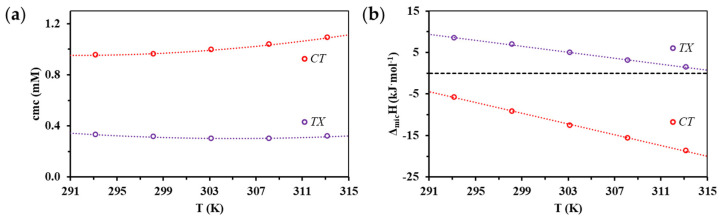
(**a**) Changes in *cmc* of CT and TX surfactants determined by ITC as a function of temperature; (**b**) Enthalpy change of micellization of CT and TX surfactants as a function of temperature.

Since, in addition to the change in composition, we also aimed to observe the effects of temperature change, the demicellization of the association colloid formed by the two compounds was studied at several temperatures. Figure 3a summarizes the temperature dependence of the *cmc* of the tested surfactants determined by ITC studies. Based on the temperature dependence, the enthalpy change accompanying micelle formation (Δ*_mic_H_vH_*) can be calculated according to the van’t Hoff equation, as shown in part (a) and (b) of Figure S2 in the Supporting Material. However, due to the nature of calorimetric measurements, the value of the (Δ*_mic_H*) can be obtained directly and more accurately from the experimental data, thus these enthalpy values are indicated in Figure 3b.

According to the result of ITC studies at the entire examined temperature range, the formation of CT micelles is exothermic, while the formation of TX micelles is the result of an endothermic process.

### 3.2. Thermodynamics (cmc, Δ_mic_G, Δ_mic_H and Δ_mic_S) of Mixed Micelle Formation

**Figure 4:**
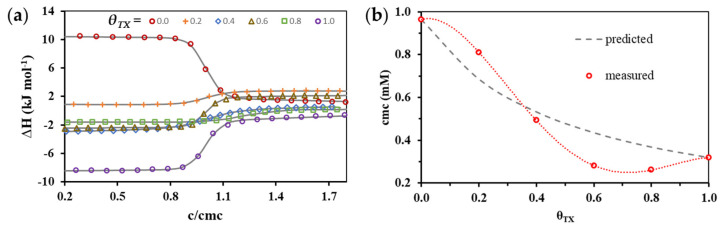
(**a**) Evolution of normalized enthalpograms from pure (*θ_TX_* = 0.0 and 1.0) and mixed (*θ_TX_* = 0.2; 0.4; 0.6 and 0.8) micelle formation at 25 °C; (**b**) Change of *cmc* values (red dots) determined from enthalpograms of pure (*θ_TX_* = 0.0 and 1.0) and mixed (*θ_TX_* = 0.2; 0.4; 0.6 and 0.8) micelle formation and calculated [29] *cmc* values (dashed line) as a function of composition of bulk phase (*θ_TX_*).

Following studies on the formation of micelles from individual CT and TX molecules, solutions were prepared using these surfactants in which the relative molecular fractions (θ) of the two surfactants varied from 0 to 1, respectively, by 0.2 units, while their concentration (ten times larger than the predicted *cmc*) was a function of ideal behavior values calculated from the theoretical work by Clint [29] (detailed explanation can be found in S.3 part of the Supplement Materials). Since, in addition to the change in composition, we also wanted to study the effect of the change in temperature, the demicellization of the association colloids formed by the two compounds and presumably of mixed composition was examined at several temperatures. In the series of measurements, ITC tests were performed at 293, 298, 303, 308 and 313 K, respectively, for all compositions (*θ_TX_* = 0.2; 0.4; 0.6 and 0.8), so after the evaluation of the 20 entalpograms, the together with data on pure components, a data set of *cmc* and a Δ*_mic_H* was available. The collective representation of the enthalpograms makes it very difficult to distinguish the data belonging to each measurement, in this way in Figure 4 only the results of the measurements performed at 298 K are presented in a representative way. In addition, for clarity, the concentration axis of Figure 4a was normalized to the enthalpograms of each composition with their respective *cmc* values, which are summarized in Table 1.

Looking at the enthalpograms summarized in Figure 4a, it can be concluded that even the appearance of small amounts of surfactants with different chemical structures dramatically changes the thermodynamic characteristics accompanying the formation of mixed micelles. The exothermic process accompanying the formation of micelles formed by CT loses the heat release character in the presence of TX. Otherwise, the endothermic property of the formation of micelles formed by pure TX is also decreasing when the ionic component appears in the mixture.

In addition, it can be stated that in aqueous solutions of pure surfactants and their mixtures the largest difference between the calculated and measured cmc values occurs when the nonionic component is present more than 50% in the mixture, as it can be seen in Figure 4b. In the case of a smaller amount the measured *cmc* value exceeds the predicted value, so we cannot identify an advantageous effect. In contrast, examination of the mixtures showed a favorable influence on the nonionic (TX) component in the 0.4 to 0.8 molar fraction range, resulting in lower micelle formation concentrations than expected. The numerical experimental data of Figure 4b are summarized in Table 1 where be-side the *cmc* and Δ*_mic_H* values and their standard deviations are also listed.

**Figure 5:**
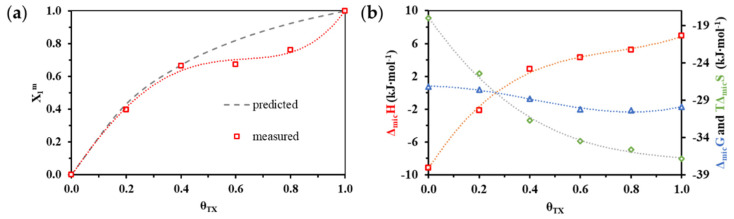
(**a**) Change of experimental (*X_1_^m^* values as red squares) and predicted (dashed line calculated by Motomura’s theory [31]) mixed micelle structure as a function of bulk phase composition (*θ_TX_*) at 298 *K*; (**b**) Variation of thermodynamic parameters (Δ*_mic_G,* Δ*_mic_H* and *T*Δ*_mic_S*) determined from enthalpograms of pure (*θ_TX_* = 0.0 and 1.0) and mixed (*θ_TX_* = 0.2; 0.4; 0.6 and 0.8) micelle formation as a function of composition of bulk phase (*θ_TX_*) at 298 K.

Having the *cmc* determined by the calorimetric measurements, the composition of the mixed micelles (*X_1_^m^*) is possible based on the calculation procedure provided by Rubingh [30] and discussed in our earliest work [17]. Figure 5a shows the change in the composition (*X_1_^m^*) of the mixed micelles as a function of the molar fraction of the nonionic component in the mixture at 298 K. In addition to the molecular fractions calculated with knowledge of the experimental *cmc* values, the gray dashed line indicates the evolution of the predicted molecular fractions assuming the ideal behavior [31] (detailed explanation can be found in S.3 part of the Supplement Materials). It can be clearly seen in Figure 5b that the composition of the mixed micelles for the *θ_TX_* = 0.6 and 0.8 surfactant ratios differ significantly from the molar fraction assuming the ideal behavior (dashed grey lines). In the micelles, the nonionic component is present in smaller amounts than expected in this region, and the enrichment of the cationic surfactant characterizes the composition of the association colloid.

Based on the results of ITC measurements the enthalpy change of micelle formation (Δ*_mic_H*) is available, while Gibbs free energy change (Δ*_mic_G*) of the association of surfactant monomers can be calculated from the *cmc* values and knowing the latter parameters, the entropy term (*T*Δ*_mic_G*) can also be calculated. Alteration of these state functions can be seen in Figure 5b as a function of composition of bulk phase (*θ_TX_*) at 298 K. The evolution of the state functions in the case of enthalpy and Gibbs free energy may suggest that an inflection point in the composition range indicates the molar ratios of outstanding significance. To demonstrate the importance of this assumption, the first derivatives of state functions are compared in the following with the results of the solubilization experiments.

### 3.3. Composition Dependence of Solubilization Capability of Mixed Micelles

**Figure 6:**
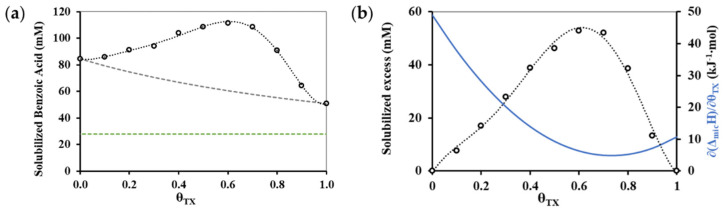
(**a**) Mixed (*θ_TX_* = 0.2; 0.4; 0.6 and 0.8) and pure (*θ_TX_* = 0.0 and 1.0) micelles solubilized amount of benzoic acid at 298 K as a function of bulk phase composition (dashed grey line indicates the ideal behavior suggested [29] solubilized amount, while dashed green line indicates the solubility of benzoic acid in water); (**b**) Change of solubilized excess (calculated from the experimental and predicted solubilized amount of benzoic acid) and the first derivative of the enthalpy change of micelle formation (∂(Δ*_mic_H*)/∂*θ_TX_*) against the bulk phase composition at 298 *K.*

Besides the calorimetric characterization of mixed micelle formation, the solubilisation capabilities of the binary surfactant mixtures were also determined for a model drug using simple acidimetric titration method. During these measurements, a surfactant mixture solution with a 100 mL volume of a surfactant mixture solution with a concentration of 0.06 M at ten different molar fractions (*θ_TX_* = 0.1; 0.2; 0.3 … 1.0) was prepared from TX and CT surfactants. Weights of 0.5 g of benzoic acid samples were measured into Erlenmeyer flasks and added to 20 mL of the respective surfactant mixture to the solid benzoic acid using automatic pipette. The solution was sonicated for 4 min to aid complete dissolution. After filtration, 5 mL of the filtrate were diluted twice with deionized water and titrated with a predetermined concentration of NaOH solution. Three replicates were made with pure (*θ_TX_* = 0.0 and 1.0) and mixed (*θ_TX_* = 0.2; 0.4; 0.6 and 0.8) surfactant solutions. The benzoic acid concentration could be determined from the volume of NaOH consumed during the titration and from the concentration ([NaOH] = 0.0959 M) of alkali solution. To calculate the solubilized benzoic acid concentration, we need to know the saturation concentration of benzoic acid in deionized water, which was also determined by titration and was found 0.0278 M. The difference between the total dissolved benzoic acid concentration and the saturation concentration gives the concentration of solubilized benzoic acid which is presented in Figure 6a as a function of composition of surfactant mixture in the bulk phase.

Experimental and calculated data of Figure 6a are summarized in Table 2 where beside the volume of alkali solution (required for neutralization) and solubilized amount of benzoic acid and standard deviations of these values are also listed. An even clearer picture emerges of the effect of the composition on solubilization ability when the solubilized excess is presented as shown in Figure 6b. The value of the excess can be obtained by calculating the solubilization capacity as stated by the ideal behavior [29], and based on the solubilization ability of the pure components. These predicted values are then subtracted from the experimentally determined data and presented in Figure 6b beside the first derivative of the enthalpy change of micelle formation (∂(Δ*_mic_H*)/∂*θ_TX_*). Figure 6b proves that the minimum of the first derivatives of enthalpy function is located at a composition that can be characterized by maximum solubilization capability.

## 4. Conclusions

Summarizing the results of the presented investigations, we can state that due to the universal nature of the isothermal titration calorimetric method, the value of critical micelle concentration (cmc) and the enthalpy change of micelle formation (Δ*_mic_H*)/∂*θ_TX_*) were successfully determined for unique surfactants as well as their mixtures. Due to this technique, we were able to determine the *cmc* of both individual surfactants and mixtures, so we had the opportunity to calculate the temperature dependence of the thermodynamic parameters and determine their standard deviation. Based on the results of ITC experiments in the investigated bulk phase mole fractions range, there are some compositions where the critical micelle concentration is lower than the ideal mixing model calculated value. Finally, we found that the equimolar binary surfactant mixtures showed higher solubilization capacity for poorly water-soluble model drugs than their individual compounds. Therefore, we can conclude that the thermodynamically beneficial compositions of mixed micelles have an advantageous property for application. Namely, they showed an enhanced ability for solubilizing a poorly water-soluble model drug. Thus, the fast and precise calorimetric analysis of mixed micelles could be a productive tool for the development of nanoscale drug carriers.

## Figures and Tables

**Table 1:** ITC determined *cmc* and Δ*_mic_H* values and their standard deviation of the mixed surfactant systems at whole composition (*θ_TX_*) range at 298 K temperature.

*θ_TX_*	*cmc* (mM)	Δ*_mic_*H (kJ mol^−1^)
0.0 ^1^	0.964 ± 0.005	−9.16 ± 0.77
0.2	0.810 ± 0.009	−2.14 ± 0.07
0.4	0.493 ± 0.003	2.92 ± 0.06
0.6	0.281 ± 0.002	4.28 ± 0.28
0.8	0.261 ± 0.002	5.22 ± 0.36
1.0 ^2^	0.319 ± 0.003	6.96 ± 0.72

^1^ Corresponds to the pure ionic (CTABr) component. ^2^ Corresponds to the pure nonionic (Triton X-100) component.

**Table 2:** Mixed micelles solubilized an amount of benzoic acid at whole composition (*θ_TX_*) range and 298 K temperature, determined by acid–base titrations.

*θ_TX_*	V*_NaOH_* (mL)	Solubilized Amount (mM)
0.0 ^1^	4.10 ± 0.10	50.8 ± 1.9
0.1	4.77 ± 0.06	63.6 ± 1.1
0.2	6.20 ± 0.10	91.0 ± 1.9
0.3	7.10 ± 0.10	108.3 ± 1.9
0.4	7.27 ± 0.06	111.5 ± 1.1
0.5	7.10 ± 0.10	108.3 ± 1.9
0.6	6.87 ± 0.06	103.8 ± 1.1
0.7	6.33 ± 0.06	93.6 ± 1.1
0.8	6.20 ± 0.10	91.0 ± 1.9
0.9	5.90 ± 0.10	85.3 ± 1.9
1.0 ^2^	5.83 ± 0.06	84.0 ± 1.1

^1^ Corresponds to the pure ionic (CTABr) component. ^2^ Corresponds to the pure nonionic (Triton X-100) component.

## Data Availability

The data presented in this study are available on request from the corresponding author. All of the raw data are not presented due to their type and large number.

## Supplementary Materials

The following are available online at https://www.mdpi.com/article/10.3390/nano11123288/s1, Figure S1: Calculation of the initial concentration (cmc) and enthalpy change (Δ*_mic_H*) attributable to micelle formation knowing the parameters (*A*_1_–*A*_6_) of the Boltzmann equation fitted to the experimental enthalpogram, Figure S2. (a) Natural-based logarithm of *cmc* of CT and TX surfactants as a function of temperature to form the van ‘t Hoff representation; (b) Changes in the micellization enthalpy of CT and TX surfactants as a function of temperature as determined by ITC studies (Δ*_mic_H* red and lilac circles) and by the temperature dependence of *cmc* (Δ*_mic_H_vH_* red and lilac diamonds), Table S1. ITC determined *cmc* and Δ*_mic_H* values and their standard deviation of the nonionic (TX) surfactant at different temperatures, Table S2. ITC determined *cmc* and Δ*_mic_H* values and their standard deviation of the cationic (CT) surfactant at different temperatures.

## Appendix A

*θ*_1_ = *n*_1_/(*n*_1_ + *n*_2_), (A1)

where *θ*_1_ is the mole fraction of the material arbitrarily chosen as the first surfactant and *n*_1_ and *n*_2_ are the amounts of the two surfactants in the two-phase and three-component systems. Thus, it is necessary that *θ*_1_ + *θ*_2_ = 1 and *X*_1_ + *X*_2_ + *X*_3_ = 1 correlations are always fulfilled. Since surfactants can adsorb in the interfacial layer and to form associates in the bulk phase, it is worthwhile to define mole fractions to determine the composition of the adsorption layer and micelles. In the adsorption layer enriched in the interfacial layer, the molar fraction of surfactant 1 is *X*_1_^σ^, which is defined by *n*_1_^σ^ and *n*_2_^σ^, respectively, while *X*_1_^m^ is determined by the molar fraction characterizing the composition of the micelle, which is determined by *n*_1_^m^ and *n*_2_^m^.

By examining a surfactant solution, the system can be divided into two phases (air and water) and two components (water and dissolved surfactant). The introduction of the two phases is necessary because, due to the directed location of the detergent at the interface, the air also becomes the determining phase. If only these two components are present, the composition of the solution can be described by two parameters: the molar fraction of water (*X*_1_) and the surfactant (*X*_2_). When a second surfactant is added to the solution, the system must also be characterized by two phases, but with three components: *X*_1_, *X*_2_ and the molar fraction of the other surfactant, i.e., *X*_3_. Since in this case *X*_1_ is much larger than *X*_2_ and *X*_3_, it is more fortunate to use the relative molar fractions of the two surfactants to characterize the composition of the “surfactant mixture” in the following context, according to the following equation.
