# Supplementary material for: Detailed Calorimetric Analysis of Mixed Micelle Formation from Aqueous Binary Surfactants for Design of Nanoscale Drug Carriers
Source: Nanomaterials (Basel). 2021 Dec 3;11(12):3288. doi: 10.3390/nano11123288 (PMC8703498; doi:10.3390/nano11123288)
Supplement: Supplementary file 1 [file nanomaterials-11-03288-s001.zip › nanomaterials-1481212-supplementary.pdf]

nanomaterials

Supplement Materials

Detailed Calorimetric Analysis of Mixed Micelle Formation From Aqueous Binary Surfactants for Design of Nanoscale Drug Carriers

Ádám Juhász [1,2], László Seres [1] Norbert Varga[1], Ditta Ungor[1], Marek Wojnicki[3] and Edit Csapó [1,2,*]

[1] MTA-SZTE Lendület "Momentum" Noble Metal Nanostructures Research Group, Interdisciplinary Excellence Center, Department of Physical Chemistry and Materials Science, Faculty of Science and Informatics, University of Szeged, Rerrich Béla sqr. 1, H-6720 Szeged, Hungary; juhaszad@chem.u-szeged.hu (Á.J.); sereslaci8@gmail.com (L.S.); vargano@chem.u-szeged.hu (N.V.); ungord@chem.u-szeged.hu (D.U.)
[2] MTA-SZTE Biomimetic Systems Research Group, Department of Medical Chemistry, University of Szeged, Dóm sqr. 8, H-6720 Szeged, Hungary
[3] Faculty of Non-Ferrous Metals, AGH University of Science and Technology, Mickiewicza Ave. 30, 30-059 Krakow, Poland; marekw@agh.edu.pl (M.W.)
* Correspondence: juhaszne.csapo.edit@med.u-szeged.hu; Tel.: +36-62-544-476

Citation: Juhász, Á.; Seres, L.; Varga, N.; Ungor, D.; Wojnicki, M.; Csapó, E. Detailed Calorimetric Analysis of Mixed Micelle Formation from Aqueous Binary Surfactants for Design of Nanoscale Drug Carriers. *Nanomaterials* **2021**, *11*, 3288. https://doi.org/10.3390/nano11123288

Academic Editor: Enrico Ferrari

Received: 10 November 2021
Accepted: 1 December 2021
Published: 3 December 2021

Publisher's Note: MDPI stays neutral with regard to jurisdictional claims in published maps and institutional affiliations.

Copyright: © 2021 by the authors. Licensee MDPI, Basel, Switzerland. This article is an open access article distributed under the terms and conditions of the Creative Commons Attribution (CC BY) license (https://creativecommons.org/licenses/by/4.0/).

S.1. Evolution of enthalpograms

Based The evolution of enthalpograms was carried out by a two-phase methodology that uses estimates from the first numeric method as starting values for the second step comprising a nonlinear parameter estimation procedure, as described in the next sentences. At the first phase, for the initial estimation of the *cmc*, the numeric derivative is calculated as the slope between each two consecutive heat effects of the injection. This derivative curve is then smoothed with a moving average, and the extremum of the smoothed function is taken as the initial value of cmc, as illustrated by the continuous orange line in **Figure S1 (a)**. In addition, the coefficients of the baselines of pre- and post-micellar stages are initially estimated based on the fit of the linear least squares to the experimental data ranges before and after the *cmc*, as the dotted orange lines in the **Figure S1 (a)** aim to display. This can be done by varying the widths of the pre- and post-transition ranges and keeping the coefficients from the best linear fits based on the smallest standard errors of the slope. However, for very shallow sigmoidal curves, this procedure is not robust enough; in these cases, the initial baselines are instead estimated based on the average value of data ranges corresponding to the initial and last 5 - 10 % of all data points.

Modelling of calorimetric enthalpograms can be performed using a modified form of sigmoidal Boltzmann equation and applying it by nonlinear regression. The following modified Boltzmann relation (equation **S1**) was used to improve the precision of the determination the *cmc* and $\Delta_{mic}H$

$$\Delta_{dil}H = \{[\ A_1 \cdot c] / [1+exp\ ((c - A_4)/A_3)]\} + A_5 \cdot c + A_6, \tag{S1}$$

where the A_i coefficients are fitting parameters. The nominator and the second term in the right-hand side of equation **S1** account for the drift of the baseline and the plateau of the sigmoid; A_3 is the width of the *cmc* transition; A_4 denotes the inflection point (where $c = cmc$); and $-(A_1 \cdot A_4 + A_2)$ measures the step height at the *cmc* which is equivalent to $\Delta_{mic}H$. In all iterative computations, convergence was detected and equation **S1** sufficiently fitted the whole experimental data.

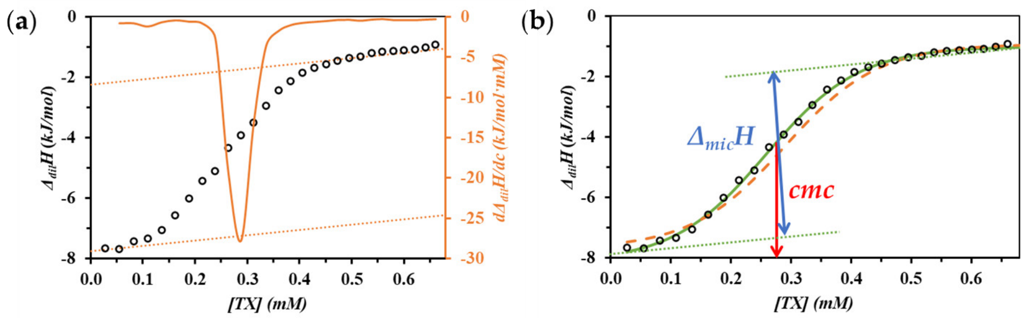

Figure S1. Calculation of the initial concentration (*cmc*) and enthalpy change ($\Delta_{mic}H$) attributable to micelle formation knowing the parameters ($A_1 - A_6$) of the Boltzmann equation fitted to the experimental enthalpogram.

The model curve generated with the initial parameter set is presented with a dashed orange line in the **Figure S1 (b)**. However, this curve approximates the experimental data well, it does not describe it with sufficient accuracy. Therefore, more accurate modeling should be performed by nonlinear regression. During regression process, the model curve is approximated to the experimental data by varying the values of the A_i coefficients and minimizing the sum of squares of the deviation ($\sum(\Delta_{dil}H_{experimental} - \Delta_{dil}H_{calculated})^2$). After the successful fit, we obtained the function indicated by a solid green line in the **Figure S1 (b)**, knowing its parameters, the value of the *cmc* and the $\Delta_{mic}H$ that can be assigned to the best fit can be calculated by based on the geometric considerations outlined in the **Figure S1 (b)** as detailed above.

S.2. Thermodynamic analysis of calorimetric data

Figure S2 (a) shows the temperature dependence of the *cmc* of the tested surfactants determined based on ITC studies, more specifically, the natural-based logarithm of *cmc* as a function of temperature to form the van 't Hoff representation. When the tangents of each curve section of the function illustrated in **Figure S2 (a)** are fitted, the value of $\Delta_{mic}H_{vH}$ can be calculated from the slope of the lines based on Equation S2

$$\Delta_{mic}H_{vH} = -R \cdot T2 \cdot \{[d(lncmc)]/dT\}_p, \quad \text{(S2)}$$

where the empirical relationship between *cmc* and temperature can be described by a quadratic polynomial ($lncmc = c + bT + aT^2$) and the parameters (a, b and c) of polynomial can be determined by fitting the polynomial to experimental data. Based on the change in the slope of the tangents, the formation of micelles can be the result of either endothermic ($\Delta_{mic}H_{vH} > 0$), exothermic ($\Delta_{mic}H_{vH} < 0$) or atermic ($\Delta_{mic}H_{vH} = 0$) processes. The minimum nature of the temperature dependence of *cmc* presumably stems from the duality that in aqueous solutions the polar part of the amphipathic molecule is well hydrated, while the interaction of its apolar part with water molecules is much more unfavorable.

Figure S2 (b) summarizes the calculated ($\Delta_{mic}H_{vH}$) and measured ($\Delta_{mic}H$) values of the enthalpy change accompanying the micelle formation of the tested amphipathic molecules, whose temperature dependence shows that at its extreme points, the thermal signature of micelle formation changes. In contrast, based on the values derived more directly from the experimental data ($\Delta_{mic}H$), it is still true for the entire examined temperature range that the formation of CT micelles is exothermic, while the formation of TX micelles is the result of an endothermic process.

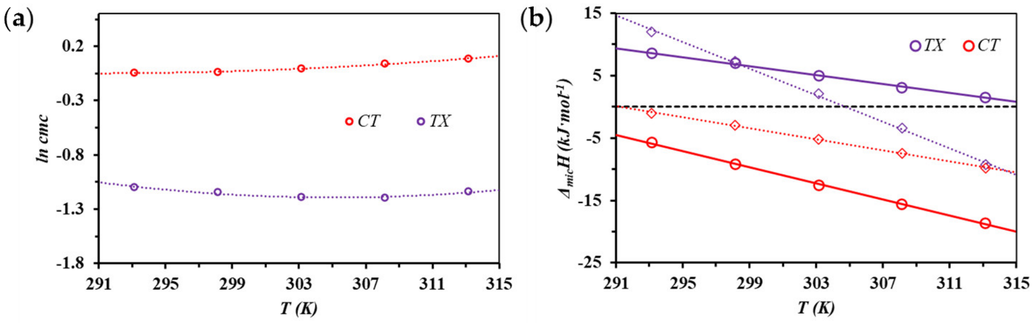

Figure S2. (a) Natural-based logarithm of *cmc* of CT and TX surfactants as a function of temperature to form the van 't Hoff representation; **(b)** Changes in the micellization enthalpy of CT and TX surfactants as a function of temperature as determined by ITC studies ($\Delta_{mic}H$ red and lilac circles) and by the temperature dependence of *cmc* ($\Delta_{mic}H_{vH}$ red and lilac diamonds)

Table S1 summarizes the measured *cmc* and $\Delta_{mic}H$ values and their standard deviation of the nonionic (TX) surfactant, while **Table S2** contains correspondent data for the cationic (CT) surfactant.

Table S1. ITC determined *cmc* and $\Delta_{mic}H$ values and their standard deviation of the nonionic (TX) surfactant at different temperatures.

T **(K)**	***cmc*** **(mM)**	$\Delta_{mic}H$ **(kJ·mol⁻¹)**
288	0.339 ± 0.004	9.91 ± 1.64
293	0.333 ± 0.003	8.55 ± 1.18
298	0.319 ± 0.003	6.96 ± 0.72
303	0.304 ± 0.003	5.03 ± 0.91
308	0.302 ± 0.004	3.08 ± 0.62
313	0.320 ± 0.007	1.53 ± 0.95

Table S2. ITC determined *cmc* and $\Delta_{mic}H$ values and their standard deviation of the cationic (CT) surfactant at different temperatures.

T **(K)**	***cmc*** **(mM)**	$\Delta_{mic}H$ **(kJ·mol⁻¹)**
288	0.955 ± 0.008	-2.93 ± 0.21
293	0.957 ± 0.005	-5.72 ± 0.42
298	0.964 ± 0.005	-9.16 ± 0.77
303	0.997 ± 0.004	-12.54 ± 1.18
308	1.038 ± 0.004	-15.65 ± 1.08
313	1.091 ± 0.004	-18.64 ± 1.38

S.3. Calculation of ideal mixed critical micelle concentration (cmc_{id}) and ideal mixed micelle composition (X_1^{id})

In the case of ideal solution theory (IST) which describes the mixing of the surfactants, the phase separation model can be used to calculate the critical micell concentration (*cmc*) of the ideal mixture (cmc_{id}) from the individual *cmc* values, cmc_1, and cmc_2, and the respective mole fractions of surfactants (θ_1 *and* θ_1). Based on the theoretical work by Clint the cmc^{id} can be calculated using the following fundamental equation:

$$1/cmc_{id} = (\theta_1/ cmc_1) + (\theta_2/ cmc_2) = (\theta_1/ cmc_1) + ((1-\theta_1)/ cmc_2) \quad \text{(S3.1)}$$

Thus, having individual cmc values, the mixed cmc value assuming ideal behaviour can be calculated for any arbitrary surfactant mixing ratio (θ_1) as illustrated by a dashed grey line in **Figure 4. B.** In addition, the ideal behavioural approach provides an opportunity to estimate the composition of ideal mixed micelles (X_1^{id}) according to Motomura's theory using the following equation:

$$X_1^{id} = (\theta_1 / cmc_2) / ((\theta_1 / cmc_2) + ((1-\theta_1)\ cmc_1))) \quad \text{(S3.2)}$$

In this way, based on the individual *cmc* values, the composition of mixed micelles assuming ideal behaviour can be predicted for any arbitrary surfactant mixing ratio (θ_1) as demonstrated by a dashed grey line in **Figure 5A**.
